# (*E*)-1-Nitro-4-(2-nitro­ethen­yl)benzene

**DOI:** 10.1107/S1600536809042561

**Published:** 2009-10-23

**Authors:** Lin-Hai Jing

**Affiliations:** a School of Chemistry and Chemical Engineering, China West Normal University, Nanchong 637002, People’s Republic of China

## Abstract

The asymmetric unit of the title compound, C_8_H_6_N_2_O_4_, consists of two independent mol­ecules with similar geometries, each adopting a *trans* configuration about the olefinic double bond. The two mol­ecules are both almost planar (r.m.s. deviations = 0.034 and 0.035 Å) and form a dihedral angle of 83.62 (2)°. Short N⋯O contacts [2.834 (3)–2.861 (3) Å] are observed between the nitro groups of neighbouring mol­ecules, with the O atom located directly atop the *p* orbital of the N atom. In the crystal, the mol­ecules are linked into a three-dimensional network by the N⋯O inter­actions and by C—H⋯O hydrogen bonds.

## Related literature

For general background to β-nitro­olefins, see: Barrett & Graboski (1986 ▶). For the synthesis, see: Valdes *et al.* (2007 ▶).
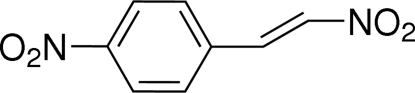


## Experimental

### 

#### Crystal data

C_8_H_6_N_2_O_4_
*M* *_r_* = 194.15
Orthorhombic, 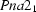

*a* = 15.847 (4) Å
*b* = 4.9991 (11) Å
*c* = 20.495 (5) Å
*V* = 1623.6 (7) Å^3^
*Z* = 8
Mo *K*α radiation
μ = 0.13 mm^−1^
*T* = 93 K
0.43 × 0.17 × 0.17 mm

#### Data collection

Rigaku AFC10/Saturn724+ diffractometer
Absorption correction: none
12157 measured reflections
1921 independent reflections
1821 reflections with *I* > 2σ(*I*)
*R*_int_ = 0.032

#### Refinement

*R*[*F* ^2^ > 2σ(*F* ^2^)] = 0.035
*wR*(*F* ^2^) = 0.090
*S* = 1.13
1921 reflections
253 parameters
1 restraint
H-atom parameters constrained
Δρ_max_ = 0.26 e Å^−3^
Δρ_min_ = −0.17 e Å^−3^

### 

Data collection: *RAPID-AUTO* (Rigaku, 2004 ▶); cell refinement: *RAPID-AUTO*; data reduction: *RAPID-AUTO*; program(s) used to solve structure: *SHELXS97* (Sheldrick, 2008 ▶); program(s) used to refine structure: *SHELXL97* (Sheldrick, 2008 ▶); molecular graphics: *XP* in *SHELXTL* (Sheldrick, 2008 ▶); software used to prepare material for publication: *SHELXL97*.

## Supplementary Material

Crystal structure: contains datablocks global, I. DOI: 10.1107/S1600536809042561/ci2942sup1.cif

Structure factors: contains datablocks I. DOI: 10.1107/S1600536809042561/ci2942Isup2.hkl

Additional supplementary materials:  crystallographic information; 3D view; checkCIF report

## Figures and Tables

**Table 1:** Hydrogen-bond geometry (Å, °)

*D*—H⋯*A*	*D*—H	H⋯*A*	*D*⋯*A*	*D*—H⋯*A*
C2—H2⋯O5^i^	0.95	2.57	3.389 (3)	144
C5—H5⋯O6^ii^	0.95	2.43	3.234 (3)	143
C6—H6⋯O7^iii^	0.95	2.56	3.469 (3)	160
C7—H7⋯O5^i^	0.95	2.45	3.304 (3)	150
C10—H10⋯O4^iv^	0.95	2.41	3.352 (3)	174
C11—H11⋯O1^v^	0.95	2.44	3.243 (3)	142
C14—H14⋯O2^vi^	0.95	2.57	3.381 (3)	144
C15—H15⋯O2^vi^	0.95	2.44	3.296 (3)	150

Symmetry codes: (i) 
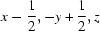
; (ii) 
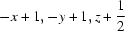
; (iii) 
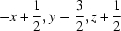
; (iv) 
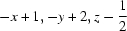
; (v) 
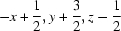
; (vi) 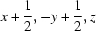.

**Fig. 1.**
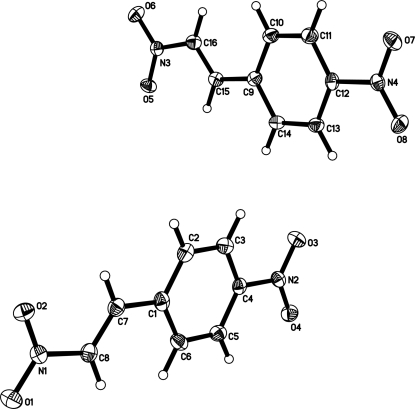
The asymmetric unit of the title compound showing the atomic numbering. Displacement ellipsoids are drawn at the 50% probability level.

## supplementary crystallographic information

### Comment

β-Nitroolefins are a class of useful and versatile building blocks in organic synthesis (Barrett & Graboski, 1986). The author reports here, the crystal structure of the title compound.

The asymmetric unit of the title compound consists of two crystallographically independent molecules (Fig. 1) each of which adopts a *trans* configuration about the olefinic double bond. All atoms in each independent molecule are almost coplanar. The N2/O3/O4 and C1-C6 (r.m.s. deviation 0.001 Å) planes form a dihedral angle of 1.4 (4)°. The dihedral angle between the C1-C6 and N1/O1/O2/C7/C8 (r.m.s. deviation 0.031 Å) planes is 2.9 (1)°. The N4/O7/O8 and  C9-C14 (r.m.s. deviation 0.004 Å) planes form a dihedral angle of 2.9 (4)°. The dihedral angle between the C9-C14 and N3/O5/O6/C15/C16 (r.m.s. deviation 0.028 Å) planes is 2.9 (1)°. The crystal packing is stabilized by C—H···O hydrogen bonds (Table 1) and N···O short contacts.

### Experimental

The title compound was synthesized according to the method reported in the literature (Valdes *et al.*, 2007). Yellow single crystals suitable for X-ray diffraction were obtained by slow evaporation of a methanol solution.

### Refinement

All H atoms were placed in calculated positions, with C-H = 0.95 Å, and refined using a riding model, with *U*_iso_(H) = 1.2*U*_eq_(C). In the absence of significant anomalous scattering, Friedel pairs were merged prior to the final refinement.

### Crystal data

**Table:**

C_8_H_6_N_2_O_4_	*F*(000) = 800
*M**_r_* = 194.15	*D*_x_ = 1.588 Mg m^−^^3^
Orthorhombic, *P**n**a*2_1_	Mo *K*α radiation, λ = 0.71073 Å
Hall symbol: P 2c -2n	Cell parameters from 5000 reflections
*a* = 15.847 (4) Å	θ = 3.3–27.5°
*b* = 4.9991 (11) Å	µ = 0.13 mm^−^^1^
*c* = 20.495 (5) Å	*T* = 93 K
*V* = 1623.6 (7) Å^3^	Prism, yellow
*Z* = 8	0.43 × 0.17 × 0.17 mm

### Data collection

**Table:**

Rigaku AFC10/Saturn724+ diffractometer	1821 reflections with *I* > 2σ(*I*)
Radiation source: Rotating Anode	*R*_int_ = 0.032
graphite	θ_max_ = 27.5°, θ_min_ = 3.3°
Detector resolution: 28.5714 pixels mm^-1^	*h* = −20→20
multi–scan	*k* = −6→6
12157 measured reflections	*l* = −20→26
1921 independent reflections	

### Refinement

**Table:**

Refinement on *F*^2^	Primary atom site location: structure-invariant direct methods
Least-squares matrix: full	Secondary atom site location: difference Fourier map
*R*[*F*^2^ > 2σ(*F*^2^)] = 0.035	Hydrogen site location: inferred from neighbouring sites
*wR*(*F*^2^) = 0.090	H-atom parameters constrained
*S* = 1.13	*w* = 1/[σ^2^(*F*_o_^2^) + (0.0583*P*)^2^ + 0.0124*P*] where *P* = (*F*_o_^2^ + 2*F*_c_^2^)/3
1921 reflections	(Δ/σ)_max_ = 0.001
253 parameters	Δρ_max_ = 0.26 e Å^−^^3^
1 restraint	Δρ_min_ = −0.16 e Å^−^^3^

### Special details

**Table:**

Geometry. All e.s.d.'s (except the e.s.d. in the dihedral angle between two l.s. planes) are estimated using the full covariance matrix. The cell e.s.d.'s are taken into account individually in the estimation of e.s.d.'s in distances, angles and torsion angles; correlations between e.s.d.'s in cell parameters are only used when they are defined by crystal symmetry. An approximate (isotropic) treatment of cell e.s.d.'s is used for estimating e.s.d.'s involving l.s. planes.
Refinement. Refinement of *F*^2^ against ALL reflections. The weighted *R*-factor *wR* and goodness of fit *S* are based on *F*^2^, conventional *R*-factors *R* are based on *F*, with *F* set to zero for negative *F*^2^. The threshold expression of *F*^2^ > σ(*F*^2^) is used only for calculating *R*-factors(gt) *etc*. and is not relevant to the choice of reflections for refinement. *R*-factors based on *F*^2^ are statistically about twice as large as those based on *F*, and *R*- factors based on ALL data will be even larger.

### Fractional atomic coordinates and isotropic or equivalent isotropic displacement parameters (Å^2^)

**Table:**

	*x*	*y*	*z*	*U*_iso_*/*U*_eq_	
O1	0.00934 (11)	−0.2104 (4)	0.37970 (9)	0.0256 (4)	
O2	0.03415 (11)	−0.1404 (4)	0.27702 (8)	0.0227 (4)	
O3	0.40990 (11)	1.2126 (4)	0.30965 (8)	0.0231 (4)	
O4	0.39197 (11)	1.1786 (3)	0.41409 (8)	0.0227 (4)	
N1	0.04305 (12)	−0.0847 (4)	0.33513 (10)	0.0181 (4)	
N2	0.37733 (12)	1.1093 (4)	0.35767 (10)	0.0172 (4)	
C1	0.20144 (14)	0.4859 (5)	0.32540 (12)	0.0201 (5)	
C2	0.24209 (16)	0.6078 (5)	0.27306 (12)	0.0212 (5)	
H2	0.2299	0.5506	0.2299	0.025*	
C3	0.29987 (15)	0.8108 (5)	0.28293 (12)	0.0194 (5)	
H3	0.3273	0.8938	0.2470	0.023*	
C4	0.31691 (14)	0.8909 (5)	0.34625 (11)	0.0170 (5)	
C5	0.27766 (15)	0.7755 (5)	0.39972 (12)	0.0195 (5)	
H5	0.2901	0.8348	0.4427	0.023*	
C6	0.21957 (15)	0.5709 (5)	0.38928 (12)	0.0214 (5)	
H6	0.1922	0.4887	0.4253	0.026*	
C7	0.14131 (14)	0.2682 (5)	0.31123 (13)	0.0200 (5)	
H7	0.1345	0.2178	0.2669	0.024*	
C8	0.09642 (15)	0.1380 (5)	0.35480 (12)	0.0198 (5)	
H8	0.0988	0.1896	0.3994	0.024*	
O5	0.69565 (11)	0.3595 (4)	0.15876 (8)	0.0214 (4)	
O6	0.72313 (10)	0.2899 (4)	0.05672 (9)	0.0234 (4)	
O7	0.33698 (11)	1.6608 (4)	0.01863 (8)	0.0239 (4)	
O8	0.31958 (11)	1.7112 (3)	0.12274 (9)	0.0222 (4)	
N3	0.68809 (12)	0.4151 (4)	0.10092 (10)	0.0167 (4)	
N4	0.35187 (12)	1.6000 (4)	0.07547 (10)	0.0167 (4)	
C9	0.52828 (13)	0.9813 (5)	0.11027 (11)	0.0170 (5)	
C10	0.51094 (15)	1.0658 (5)	0.04660 (12)	0.0203 (5)	
H10	0.5396	0.9851	0.0110	0.024*	
C11	0.45240 (14)	1.2658 (5)	0.03503 (12)	0.0204 (5)	
H11	0.4398	1.3216	−0.0082	0.024*	
C12	0.41275 (14)	1.3822 (5)	0.08803 (11)	0.0167 (5)	
C13	0.42840 (15)	1.3075 (5)	0.15165 (12)	0.0184 (5)	
H13	0.4004	1.3928	0.1870	0.022*	
C14	0.48642 (15)	1.1037 (5)	0.16264 (12)	0.0173 (5)	
H14	0.4978	1.0470	0.2060	0.021*	
C15	0.58824 (14)	0.7649 (5)	0.12470 (12)	0.0175 (5)	
H15	0.5946	0.7135	0.1691	0.021*	
C16	0.63421 (15)	0.6356 (5)	0.08075 (11)	0.0191 (5)	
H16	0.6320	0.6871	0.0362	0.023*	

### Atomic displacement parameters (Å^2^)

**Table:**

	*U*^11^	*U*^22^	*U*^33^	*U*^12^	*U*^13^	*U*^23^
O1	0.0256 (9)	0.0291 (9)	0.0220 (9)	−0.0036 (8)	0.0025 (7)	0.0041 (8)
O2	0.0238 (9)	0.0263 (10)	0.0180 (9)	0.0004 (7)	0.0000 (7)	−0.0033 (8)
O3	0.0225 (8)	0.0260 (9)	0.0209 (8)	−0.0040 (8)	0.0013 (7)	0.0046 (7)
O4	0.0261 (10)	0.0234 (9)	0.0186 (8)	−0.0004 (7)	−0.0041 (7)	−0.0025 (7)
N1	0.0155 (10)	0.0182 (10)	0.0204 (10)	−0.0008 (8)	−0.0018 (8)	0.0016 (8)
N2	0.0149 (9)	0.0170 (9)	0.0198 (9)	0.0004 (8)	−0.0016 (8)	0.0031 (8)
C1	0.0159 (11)	0.0164 (11)	0.0279 (14)	0.0038 (10)	−0.0002 (9)	0.0024 (10)
C2	0.0216 (12)	0.0197 (11)	0.0224 (12)	0.0023 (10)	−0.0031 (10)	−0.0033 (9)
C3	0.0171 (11)	0.0220 (12)	0.0191 (12)	0.0024 (9)	−0.0008 (9)	0.0019 (10)
C4	0.0131 (10)	0.0152 (10)	0.0228 (12)	0.0038 (9)	0.0001 (9)	0.0005 (10)
C5	0.0210 (11)	0.0217 (13)	0.0159 (12)	0.0028 (11)	−0.0022 (9)	0.0007 (9)
C6	0.0210 (11)	0.0216 (12)	0.0217 (12)	0.0017 (10)	0.0021 (10)	0.0060 (10)
C7	0.0199 (11)	0.0186 (12)	0.0215 (11)	0.0032 (11)	−0.0011 (10)	−0.0011 (10)
C8	0.0199 (12)	0.0184 (11)	0.0210 (11)	−0.0013 (9)	−0.0021 (9)	−0.0019 (9)
O5	0.0235 (9)	0.0239 (9)	0.0167 (8)	0.0021 (7)	−0.0017 (7)	0.0045 (7)
O6	0.0237 (9)	0.0237 (9)	0.0230 (9)	0.0051 (7)	−0.0005 (7)	−0.0066 (8)
O7	0.0249 (9)	0.0249 (10)	0.0218 (9)	0.0030 (7)	−0.0035 (7)	0.0067 (8)
O8	0.0210 (8)	0.0215 (8)	0.0241 (9)	0.0057 (7)	0.0001 (7)	−0.0039 (7)
N3	0.0147 (9)	0.0161 (9)	0.0193 (10)	−0.0008 (8)	−0.0003 (8)	0.0003 (8)
N4	0.0156 (9)	0.0149 (9)	0.0194 (10)	−0.0017 (8)	−0.0019 (7)	0.0014 (8)
C9	0.0149 (10)	0.0178 (11)	0.0184 (12)	−0.0025 (9)	0.0007 (9)	−0.0020 (9)
C10	0.0216 (12)	0.0212 (12)	0.0182 (11)	0.0032 (10)	0.0029 (9)	−0.0007 (10)
C11	0.0213 (11)	0.0220 (12)	0.0179 (12)	0.0006 (11)	−0.0023 (9)	0.0002 (9)
C12	0.0135 (10)	0.0143 (10)	0.0222 (12)	0.0017 (9)	−0.0020 (9)	0.0011 (9)
C13	0.0180 (12)	0.0195 (10)	0.0176 (11)	−0.0009 (9)	0.0032 (9)	−0.0006 (10)
C14	0.0168 (11)	0.0206 (11)	0.0146 (11)	−0.0001 (10)	0.0005 (9)	0.0000 (9)
C15	0.0157 (10)	0.0195 (12)	0.0172 (11)	−0.0007 (10)	−0.0014 (9)	0.0005 (9)
C16	0.0181 (11)	0.0210 (12)	0.0182 (11)	0.0009 (9)	−0.0015 (9)	0.0008 (9)

### Geometric parameters (Å, °)

**Table:**

O1—N1	1.231 (3)	O5—N3	1.223 (3)
O2—N1	1.231 (3)	O6—N3	1.233 (3)
O3—N2	1.225 (3)	O7—N4	1.227 (3)
O4—N2	1.229 (3)	O8—N4	1.229 (3)
N1—C8	1.455 (3)	N3—C16	1.455 (3)
N2—C4	1.471 (3)	N4—C12	1.477 (3)
C1—C2	1.392 (3)	C9—C10	1.399 (3)
C1—C6	1.406 (3)	C9—C14	1.402 (3)
C1—C7	1.476 (3)	C9—C15	1.470 (3)
C2—C3	1.382 (3)	C10—C11	1.385 (3)
C2—H2	0.95	C10—H10	0.95
C3—C4	1.385 (3)	C11—C12	1.383 (3)
C3—H3	0.95	C11—H11	0.95
C4—C5	1.386 (3)	C12—C13	1.379 (3)
C5—C6	1.392 (4)	C13—C14	1.391 (3)
C5—H5	0.95	C13—H13	0.95
C6—H6	0.95	C14—H14	0.95
C7—C8	1.314 (4)	C15—C16	1.327 (3)
C7—H7	0.95	C15—H15	0.95
C8—H8	0.95	C16—H16	0.95
			
O2—N1—O1	123.6 (2)	O5—N3—O6	123.5 (2)
O2—N1—C8	120.5 (2)	O5—N3—C16	120.3 (2)
O1—N1—C8	115.9 (2)	O6—N3—C16	116.11 (19)
O3—N2—O4	123.9 (2)	O7—N4—O8	123.8 (2)
O3—N2—C4	117.33 (19)	O7—N4—C12	118.28 (19)
O4—N2—C4	118.79 (19)	O8—N4—C12	117.89 (19)
C2—C1—C6	119.4 (2)	C10—C9—C14	119.3 (2)
C2—C1—C7	118.0 (2)	C10—C9—C15	122.5 (2)
C6—C1—C7	122.6 (2)	C14—C9—C15	118.2 (2)
C3—C2—C1	121.0 (2)	C11—C10—C9	120.6 (2)
C3—C2—H2	119.5	C11—C10—H10	119.7
C1—C2—H2	119.5	C9—C10—H10	119.7
C2—C3—C4	118.6 (2)	C12—C11—C10	118.3 (2)
C2—C3—H3	120.7	C12—C11—H11	120.9
C4—C3—H3	120.7	C10—C11—H11	120.9
C3—C4—C5	122.2 (2)	C13—C12—C11	123.2 (2)
C3—C4—N2	119.4 (2)	C13—C12—N4	118.8 (2)
C5—C4—N2	118.4 (2)	C11—C12—N4	118.0 (2)
C4—C5—C6	118.7 (2)	C12—C13—C14	118.1 (2)
C4—C5—H5	120.6	C12—C13—H13	121.0
C6—C5—H5	120.6	C14—C13—H13	121.0
C5—C6—C1	120.0 (2)	C13—C14—C9	120.6 (2)
C5—C6—H6	120.0	C13—C14—H14	119.7
C1—C6—H6	120.0	C9—C14—H14	119.7
C8—C7—C1	125.5 (2)	C16—C15—C9	125.2 (2)
C8—C7—H7	117.2	C16—C15—H15	117.4
C1—C7—H7	117.2	C9—C15—H15	117.4
C7—C8—N1	120.3 (2)	C15—C16—N3	119.9 (2)
C7—C8—H8	119.8	C15—C16—H16	120.0
N1—C8—H8	119.8	N3—C16—H16	120.0
			
C6—C1—C2—C3	0.0 (4)	C14—C9—C10—C11	−0.8 (4)
C7—C1—C2—C3	−179.2 (2)	C15—C9—C10—C11	178.3 (2)
C1—C2—C3—C4	0.2 (4)	C9—C10—C11—C12	1.0 (4)
C2—C3—C4—C5	−0.4 (4)	C10—C11—C12—C13	−0.4 (4)
C2—C3—C4—N2	−179.1 (2)	C10—C11—C12—N4	178.6 (2)
O3—N2—C4—C3	0.4 (3)	O7—N4—C12—C13	−178.4 (2)
O4—N2—C4—C3	−180.0 (2)	O8—N4—C12—C13	2.2 (3)
O3—N2—C4—C5	−178.4 (2)	O7—N4—C12—C11	2.6 (3)
O4—N2—C4—C5	1.2 (3)	O8—N4—C12—C11	−176.8 (2)
C3—C4—C5—C6	0.4 (4)	C11—C12—C13—C14	−0.5 (4)
N2—C4—C5—C6	179.2 (2)	N4—C12—C13—C14	−179.5 (2)
C4—C5—C6—C1	−0.3 (4)	C12—C13—C14—C9	0.8 (3)
C2—C1—C6—C5	0.1 (4)	C10—C9—C14—C13	−0.2 (3)
C7—C1—C6—C5	179.3 (2)	C15—C9—C14—C13	−179.3 (2)
C2—C1—C7—C8	−178.2 (2)	C10—C9—C15—C16	2.9 (4)
C6—C1—C7—C8	2.6 (4)	C14—C9—C15—C16	−178.0 (2)
C1—C7—C8—N1	−176.1 (2)	C9—C15—C16—N3	−176.7 (2)
O2—N1—C8—C7	−5.7 (3)	O5—N3—C16—C15	−4.9 (3)
O1—N1—C8—C7	173.6 (2)	O6—N3—C16—C15	173.9 (2)

### Hydrogen-bond geometry (Å, °)

**Table:**

*D*—H···*A*	*D*—H	H···*A*	*D*···*A*	*D*—H···*A*
C2—H2···O5^i^	0.95	2.57	3.389 (3)	144
C5—H5···O6^ii^	0.95	2.43	3.234 (3)	143
C6—H6···O7^iii^	0.95	2.56	3.469 (3)	160
C7—H7···O5^i^	0.95	2.45	3.304 (3)	150
C10—H10···O4^iv^	0.95	2.41	3.352 (3)	174
C11—H11···O1^v^	0.95	2.44	3.243 (3)	142
C14—H14···O2^vi^	0.95	2.57	3.381 (3)	144
C15—H15···O2^vi^	0.95	2.44	3.296 (3)	150

Symmetry codes: (i) *x*−1/2, −*y*+1/2, *z*; (ii) −*x*+1, −*y*+1, *z*+1/2; (iii) −*x*+1/2, *y*−3/2, *z*+1/2; (iv) −*x*+1, −*y*+2, *z*−1/2; (v) −*x*+1/2, *y*+3/2, *z*−1/2; (vi) *x*+1/2, −*y*+1/2, *z*.
